# Genetic engineering of T cells with receptors from NY-ESO-1-specific tumor-recognizing CD4+ T cell as a novel approach for adoptive T cell therapy

**DOI:** 10.1186/2051-1426-3-S2-P34

**Published:** 2015-11-04

**Authors:** Junko Matsuzaki, Takemasa Tsuji, Immanuel Luescher, Hiroshi Shiku, Junichi Mineno, Sachiko Okamoto, Lloyd Old, Protul Shrikant, Sacha Gnjatic, Kunle Odunsi

**Affiliations:** 1 Roswell Park Cancer Institute, Buffalo, NY, USA; 2 Ludwig Center for Cancer Research, Epalinges, Switzerland; 3 Mie University Graduate School of Medicine, Tsu, Japan; 4 TAKARA BIO INC, Otsu, Japan; 5 Ludwig Institute for Cancer Research, New York, NY, USA; 6 Mayo Clinic, Scottsdale, AZ, USA; 7 MSSM, New York, NY, USA

## Background

Tumor antigen-specific CD4^+^ T cells generally orchestrate and regulate innate and adaptive immune cells to provide immune surveillance against malignancy. However, activation of antigen-specific CD4^+^ T cells is restricted at local tumor sites where antigen-presenting cells are frequently dysfunctional, which can cause rapid exhaustion of anti-tumor immune responses. Herein, we characterize anti-tumor effects of a unique human CD4^+^ helper T cell subset that directly recognizes the cytoplasmic tumor antigen, NY-ESO-1, presented by MHC class II (MHC-II) on cancer cells. In addition, we clone the TCR gene from tumor-recognizing CD4^+^ T cells (TR-CD4) and test the function of TCR gene-engineered cells.

## Methods

NY-ESO-1-specfic CD4^+^ or CD8^+^ T cells were obtained from ovarian cancer patients who received NY-ESO-1 vaccine. Full-length TCR α and β chain genes of TR-CD4 were cloned by 5' RACE PCR. TCR gene was transduced into activated T cells by MSCV-based retroviral vector. The effector function was evaluated against cognate peptide-pulsed target cells or NY-ESO-1^+^MHC-II^+^ cancer cell lines by ELISA, intracellular cytokine staining or CTL assay.

## Results

TR-CD4, but not conventional NY-ESO-1-specific CD4^+^ T cells, directly recognized cancer cells in MHC-II-dependent and NY-ESO-1-specific manners. Presentation of intracellular NY-ESO-1 on MHC-II by cancer cells required non-classical MHC-II antigen presentation mechanisms. Upon direct recognition of cancer cells, TR-CD4 potently induced IFN-γ-dependent growth arrest in cancer cells. In addition, direct recognition of cancer cells triggers TR-CD4 to provide help to NY-ESO-1-specific CD8^+^ T cells by enhancing cytotoxic activity, and improving viability and proliferation. Notably, the TR-CD4 either alone or in combination with NY-ESO-1-specific CD8^+^ T cells significantly inhibited tumor growth *in vivo* in a xenograft model. Finally, retroviral gene-engineering of polyclonally activated T cells with TCR derived from TR-CD4 successfully produced large numbers of functional TR-CD4.

## Conclusions

These observations provide mechanistic insights into the role of TR-CD4 in tumor immunity, and suggest that approaches to utilize TR-CD4 will augment anti-tumor immune responses for durable therapeutic efficacy in cancer patients. Large numbers of TR-CD4 that directly recognize cancer cells and enhance CD8^+^ T cell functions can be generated by gene-engineering with TCR from TR-CD4. Antigen-presenting cell-independent provision of CD4-help by TR-CD4 is especially important to enhance durable CD8^+^ T cell anti-tumor functions at the tumor local site. Adoptive T cell therapy using TR-CD4 in combination with CD8^+^ T cells could be a promising strategy for effective eradication of tumors.

